# A Low-Cost and Portable Dual-Channel Fiber Optic Surface Plasmon Resonance System

**DOI:** 10.3390/s17122797

**Published:** 2017-12-04

**Authors:** Qiang Liu, Yun Liu, Shimeng Chen, Fang Wang, Wei Peng

**Affiliations:** School of Physics and Optoelectronic Technology, Dalian University of Technology, 2 Linggong Road, Ganjingzi District, Dalian 116024, China; s201044125@mail.dlut.edu.cn (Q.L.); liuyun89@dlut.edu.cn (Y.L.); chenshimengdlut@foxmail.com (S.C.); w15122514950@163.com (F.W.)

**Keywords:** Low cost and portable, fiber optic surface plasmon resonance, dual-channel, biosensor

## Abstract

A miniaturization and integration dual-channel fiber optic surface plasmon resonance (SPR) system was proposed and demonstrated in this paper. We used a yellow light-emitting diode (LED, peak wavelength 595 nm) and built-in web camera as a light source and detector, respectively. Except for the detection channel, one of the sensors was used as a reference channel to compensate nonspecific binding and physical absorption. We packaged the LED and surface plasmon resonance (SPR) sensors together, which are flexible enough to be applied to mobile devices as a compact and portable system. Experimental results show that the normalized intensity shift and refractive index (RI) of the sample have a good linear relationship in the RI range from 1.328 to 1.348. We used this sensor to monitor the reversible, specific interaction between lectin concanavalin A (Con A) and glycoprotein ribonuclease B (RNase B), which demonstrate its capabilities of specific identification and biochemical samples concentration detection. This sensor system has potential applications in various fields, such as medical diagnosis, public health, food safety, and environment monitoring.

## 1. Introduction

Surface plasmon resonance (SPR) is a collective resonance of free electrons, which occurs at the interface of metal and a dielectric material [1]. It is sensitive to the refractive index (RI) change of the external environment, especially in association and dissociation reactions of biochemistry [2,3]. It has been widely used in the fields of environmental monitoring [4], food safety [5,6], disease diagnosis [7], and clinical analysis [8] for its advantages of label-free and high sensitivity. In 1990, the first SPR commercialized device based on a prism was developed by Biacore AB Company. However, due to its large volume and high cost, it was only applicable to the laboratory. In 1993, a miniature SPR sensor based on step-type optical fiber was proposed by Jorgenson RC and Yee SS [9]. Since then, fiber optic SPR sensors have been studied intensively. Compared with prism-based SPR sensors, fiber optic SPR sensors have the advantages of simple structure, low cost, small sample volume, and remote sensing applications. The major challenges of SPR biosensors lies not only in the improvement of sensitivity but also in providing an integrated, low-cost, reusable, and high-throughput biosensor. In addition, the worldwide use of mobile devices creates significant opportunities for low-cost and portable biosensors for point-of-care testing. Preechaburana [10] demonstrated an angle-resolved surface plasmon resonance sensor which can be applied to different cell phones, as the display of the cell phone served as a light source and the font camera served as a detector. However, the optical coupler was put on the cell phone screen, which affects its normal use during detection. Bremer et al. demonstrated a smartphone-based fiber optic glycerol SPR sensor [11] that uses a Thorlabs holographic reflective diffraction grating with 1200 lines/mm to disperse the light into a line spectrum; as an mp4-video (480 × 640 pixels) was taken and subsequently processed by Matlab, it was not able to realize real-time analysis. Besides, the sensor was coated with a silver layer, which is easily oxidized. Considering some noise-causing scenarios in biosensing such as temperature variations, non-specific binding, and instrumental instability, a reference channel is necessary for SPR biosensors [12,13,14]. Most of the reported mobile device-based SPR sensors have complex optical configurations and are not equipped with a reference channel for self-compensation [10,11,15]. 

**Figure 1:**
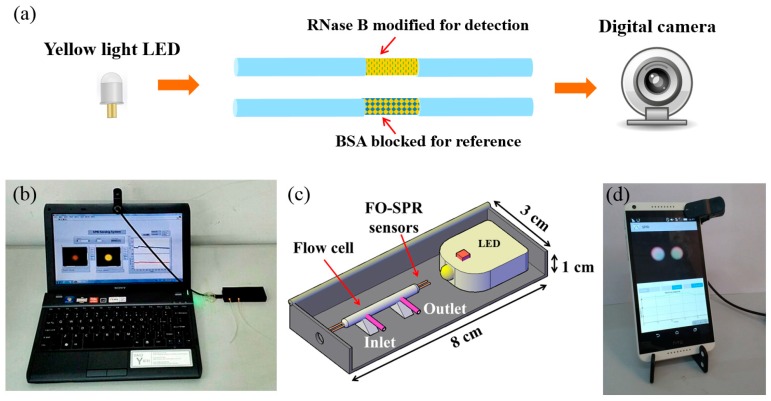
Dual-channel fiber optic SPR system. (**a**) Schematic of the sensor system, (**b**) Laptop-based application, (**c**) Diagram of components inside the sensing box, (**d**) Smartphone-based application.

In this paper, we propose a flexible compact dual-channel SPR system. Figure 1 illustrates the designed sensor system and its applications schematically. As shown in Figure 1a, a yellow light LED with 590 nm emission wavelength served as the light source, and a web camera served as the detector. SPR sensors are fabricated by a 50-nm Au layer coating on the surface of an optic fiber, which provides a stable performance and renders them easy to be fabricated. One of the sensors served as a reference channel to compensate nonspecific binding and physical absorption. Compared with our previous work on smartphone-based SPR sensors [16], the current system lowers the cost and simplifies the optical configuration, making it flexible enough for different types of mobile device platforms such as laptops (Figure 1b), smartphones (Figure 1d), and tablets. The bulk refractive index sensing of the system indicates that the system effectively compensates for background RI variations, and the reference channel is blocked by bovine serum albumin, which can compensate for non-specific binding more effectively. Herein, its application on a laptop was employed as an example, and real-time sensing results could be displayed on the screen of the laptop through the custom LabVIEW software. The lab-scale experiment shows that the system has good performance in detecting the concanavalin A (Con A) samples. The size of the biosensor is small enough to be put in the pocket for field applications. With the growing demand for convenient biosensors, it will have great potential in the fields of medical diagnosis, public health, and environment monitoring.

## 2. Materials and Methods 

### 2.1. System Design and Setup 

Gold (Au) and silver (Ag) are mainly used for fabricating the metal film of SPR sensors. However, Ag metal-based SPR sensors are not chemically stable because Ag metal is very prone to oxidation. SPR sensors coated with an Au layer have stable performance and are easy to fabricate. Considering the lifetime and robustness of the sensor, we chose gold metal as the plasmonic material. We used a kind of plastic cladding silica optical fiber with a numerical aperture of 0.37 (Hard polymer cladding optical fiber (HPOF), HP 400/430-37/730E, YOFC Inc., Wuhan, China) to fabricate the sensor in this work. The diameters of the fiber core and cladding were 400 and 430 μm respectively. The sensor had a length of 4 cm, and a 6-mm sensing part without cladding and coated with a 50-nm Au layer in the middle of the fiber.

The sensor contained a black box for holding elements (8 cm (length) × 3 cm (width) × 1 cm (height)), a connecting line (30 cm) with two optical fibers inside for signal transmission, and a laptop (PCG-31311T, Sony Corporation, Tokyo, Japan) for data acquisition, processing, and results display. The box was made from polycarbonate with a black color to exclude interference from stray light. There were two components inside the box including a yellow light LED and the SPR sensors packaged with a flow cell. To realize relatively stable sample injection and liquid flow, a mini pump was used for sample injection with a flow rate of 250 μL/min. Sample solutions flowed into the flow cell from the inlet and flowed out from the outlet.

### 2.2. Principle for Real-Time Detection

**Figure 2:**
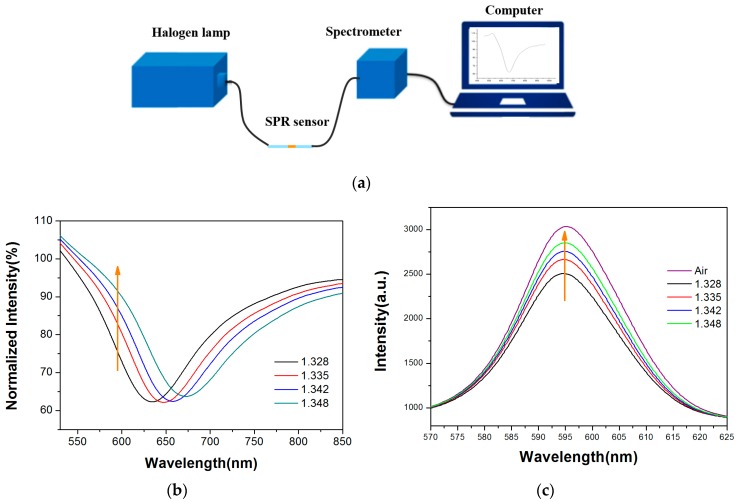
Spectral characteristics of fiber optic SPR sensor. (**a**) Wavelength interrogation system used to verify the fabricated fiber optic SPR sensors; (**b**) Normalized spectrums of the fiber optic SPR sensor in different RIs (refractive indices); (**c**) Transmission spectrums in air and sodium chloride solutions with different RIs using yellow light LED (light-emitting diode) as light source.

SPR refers to the coupling resonance between the p-polarized (TM polarized) light and surface plasmon wave on the metal film that occurs on the metal-dielectric interface. The resonance can lead to a rapid reduction of intensity of the reflected light, which lead to a resonate dip in spectrum. Because the SPR resonance dip is sensitive to external RI change, we can measure its RI sensitivity by detecting SPR resonance wavelength changes through wavelength interrogation. To explain the principle and verify the performance, we used a wavelength interrogation SPR system as shown in Figure 2a, which consists of a halogen lamp (HL-2000, Ocean Optics, FL, USA) and a high-resolution spectrometer (HR4000, Ocean Optics, FL, USA). The fabricated sensor was connected to the devices using SMA905 connectors. In an SPR-based fiber optic sensor, because all the guided rays are launched, the detector obtains the accumulation of all transmission modes corresponding to each angle. Figure 2b shows normalized spectrums of the sensor in different RIs; the sensor shows a clear resonance which shifts towards the red wavelength range when the RI increases in the surrounding liquid, which is consistent with the reported SPR sensors based on Au coatings [17]. We changed the liquid RIs by using different sodium chloride solutions which were calibrated by an Abbe refractometer (WAY-2S). The testing results show that this sensor has a linear response in an RI range of 1.328–1.357 with a sensitivity of 2223.6 nm/RIU (Refractive index unit).

The intensity at a fixed wavelength will change along with the SPR curve shifting caused by variations of RI. By monitoring the intensity of two wavelengths at the band of dips, it can be seen that the intensity of the shorter wavelength increases and that of the longer wavelength decreases. When a particular sample solution flowed across the functionalized SPR sensing layer, bio-specific interaction occurred on the gold film and the RI increased. Therefore, SPR curve shifting caused by bio-specific interaction can be monitored by measuring light intensity variations at a fixed wavelength. To obtain a positive response to the RIs, we set the monitoring wavelength location on the left side of the dips. In this work, a yellow light LED (595 nm emission wavelength, 20 nm bandwidth) was selected as the light source without using a narrowband filter, which thus reduced the system’s costs. Transmission spectra (obtained by the HR4000 spectrometer, Ocean Optics, FL, USA) of the sensor in air condition and in sodium chloride solutions with different RIs are shown in Figure 2c. Consistent with the results shown in Figure 2b, the intensity at the wavelength around 595 nm has a positive response to the variations of RI.

Low-cost and real-time monitoring can be easily realized by mobile devices equipped with a digital camera. The image acquisition and processing functions can be completed through the built-in web camera and the LabVIEW software without an expensive frame grabber or digitizer. The real-time image acquisition and processing program was based on LabVIEW IMAQ Vision. This software combines the merits of both LabVIEW and IMAQ Vision, which have a graphical programming environment and rich image processing functions [18]. The program captures an image of the light spots once per second. After drawing the regions of interest (ROIs) for the two light spots separately, it extracts the intensity color plane and calculates the average intensity value. Intensity-time coordinates of the two channels were displayed on the screen in real time. Based on the same principle, it can be applied to different mobile platforms by simple calibration, which makes this dual-channel SPR system much more flexible.

### 2.3. Biodetection Assay

**Figure 3:**
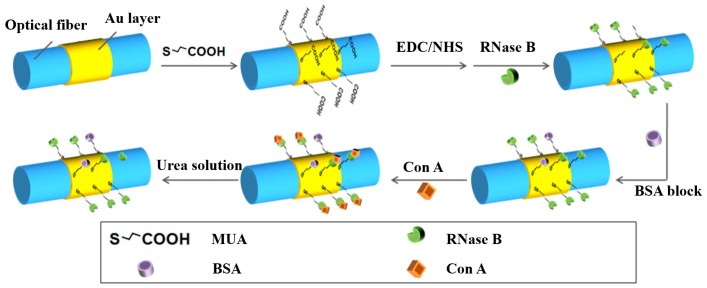
Functionalization and detection procedure of the SPR sensor.

The favorable interaction between alpha-mannose-presenting glycoprotein ribonuclease B (RNase B) and alpha-mannose-specific lectin Con A has been reported in solution [19] and in the adsorbed state [20]. As a representative example, we demonstrated this dual-channel SPR system through monitoring reversible, specific interaction between the lectin Con A and glycoprotein ribonuclease B (RNase B). The SPR sensor was functionalized with an amine-coupling reaction to facilitate the attachment of RNase B (as shown in Figure 3). The sensing region of SPR sensors was soaked in 1 mmol/L mercaptoundecanic acid (MUA) in ethanolic for 12 h to form a carboxyl surface and then immersed in ultrapure water that contained 0.5 mol/L *N*-hydroxysuccinimide (NHS) and 0.55 mol/L 1-ethyl-3-(3-dimethylamino-propyl) carbodiimide hydrochloride (EDC) at 4 °C for 30 min to convert the carboxyl surface to activated ester. The treated sensors were dipped in RNase B (0.1 mg/mL in 0.01 mol/L phosphate buffered solution (PBS; pH = 7.4)) for 30 min. Unreacted ester groups were blocked by bovine serum albumin (BSA, 1 mg/mL in PBS) for 15 min. The SPR sensor used as reference channel was blocked by BSA without the attachment of RNase B. Actual response for the binding of Con A can be corrected by the difference between the detection channel and the reference channel.

## 3. Results and Discussion

### 3.1. Bulk Refractive Index Sensing

**Figure 4:**
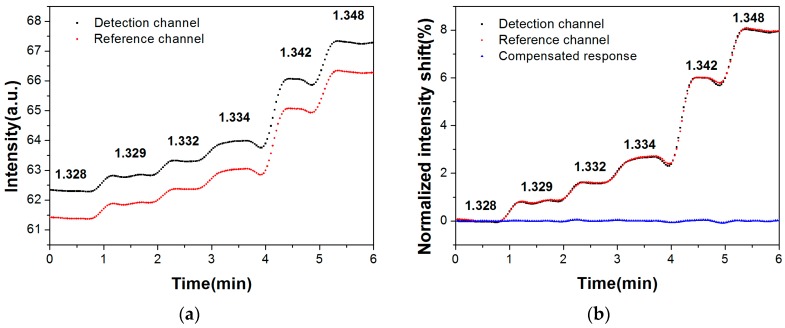

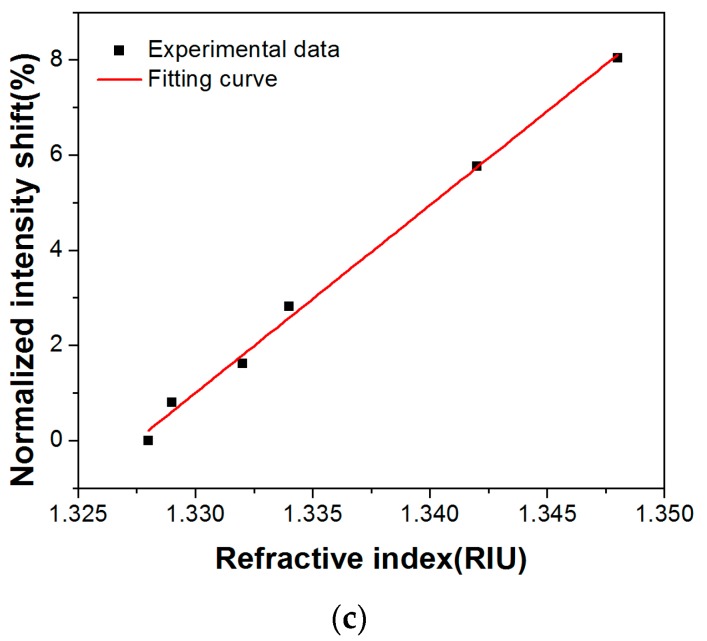
Real-time responses of the dual-channel SPR system upon sequential injection of sodium chloride solutions at increasing refractive index (1.328–1.348). (**a**) Original responses; (**b**) Normalized responses and compensated responses; (**c**) The fitting curve established by normalized intensity shift with different RIs.

Sodium chloride solutions with different RIs were used to validate the performance of the SPR system. Original real-time responses of the detection channel and reference channel upon sequential injection of sodium chloride solutions at increasing RI (1.328–1.348) are shown in Figure 4a. The intensity of the detection channel and reference channel were normalized at the beginning in an aqueous environment. With the stepwise increase of RI of the injected samples, the normalized responses of both channels showed step-up trends. We found that the RI sensitivities of two channels were at the same level with acceptable slight error. Figure 4b shows that the compensated responses come to be a straight line with a mean of about zero, which indicates that the self-referenced dual-channel SPR system effectively compensated for background RI variations.

Figure 4c illustrates the linear relationship between the normalized intensity shift and the RIs in the range of 1.328–1.348. The system achieved a RI sensitivity of 394.56%/RIU. Considering the stability in time responses, the RI limit of detection (LOD) was calculated to be 4.3 × 10^−4^ RIU, which is almost same as that of a commercial Abbe refractometer. The sensitivity and resolution of the dual-channel SPR system were determined by sensitive wavelength response and the narrow full width at half maximum (FWHM) of sensors, the high performance of camera, narrow bandwidth of light source, stable injection device, etc. Therefore, the system can be improved to be a highly sensitivity-stabilized multi-channel SPR system to work for different field applications.

### 3.2. Biodetection of Con A

**Figure 5:**
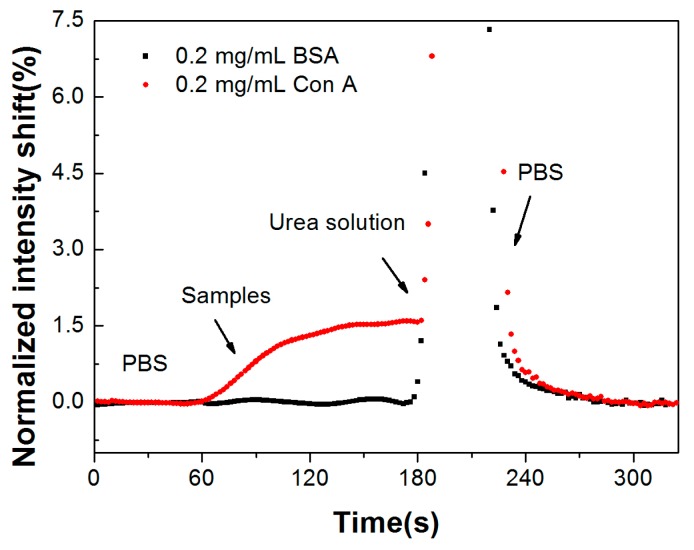
Detection of specific binding between RNase B and Con A and non-specific binding of BSA (bovine serum albumin).

A mini pump was used for sample injection, with a flow rate of 250 μL/min. As shown in Figure 5, PBS buffer was pumped into the flow cell to obtain the baseline. Then, 500 μL Con A sample was pumped into the flow cell when the baseline was stable. To regenerate the sensing region for next detection cycle of the Con A sample, 8.0 mol/L urea solution was used. The specific binding of Con A caused an obvious intensity shift, and no obvious response could be seen for the 0.2 mg/mL BSA sample, which indicates that the sensors might have good performance in complex sample detection without a purification procedure.

**Figure 6:**
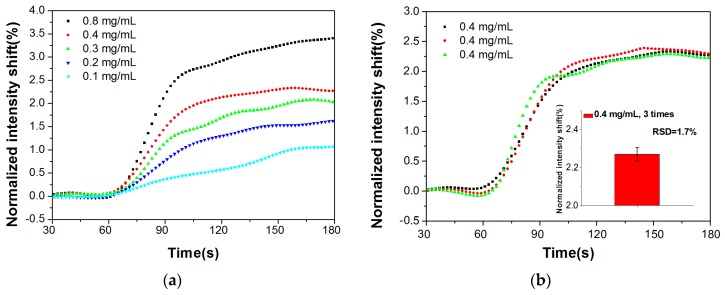
Con A samples detection. (**a**) Real-time response curves of detecting Con A samples, (**b**) Reproducibility of detecting 0.4 mg/mL Con A samples for three independent tests.

We used the system to detect different concentrations of Con A samples, ranging from 0.1 mg/mL to 0.8 mg/mL, the corrected real-time response is shown in Figure 6a. To evaluate the sensor stability, 0.4 mg/mL Con A samples were tested three times, and the relative standard deviation of the detection values was about 1.7% (Figure 6b). As shown above, this biodetection assay is capable of the specific identification and concentration detection of biochemical samples. We can further enhance its sensitivity and resolution by optimizing the performances and utilizing secondary antibodies or nanoparticles to achieve signal amplification [21,22,23].

## 4. Conclusions

We demonstrated a compact and portable dual-channel fiber optic SPR sensor system in this paper. Simple optical configuration renders it flexible enough to be applied to different mobile devices. The normalized intensity shift and RI value of the sample showed a good linear relationship in the RI range from 1.328 to 1.348. Its capability for biodetection applications is demonstrated by the concentration detection of Con A. This SPR sensor can be implemented in general environments with further investigations, providing a universal and promising platform for cost-effective biosensors.
